# Pregnancy, thyroid, and the potential use of selenium

**DOI:** 10.1007/s42000-019-00144-2

**Published:** 2019-11-13

**Authors:** Alicja Hubalewska-Dydejczyk, Leonidas Duntas, Aleksandra Gilis-Januszewska

**Affiliations:** 1 Chair and Department of Endocrinology, Jagiellonian University Medical College, Kopernika 17 Str, 31-501 Cracow, Poland; 2 Unit of Endocrinology, Diabetes and Metabolism, Evgenideion Hospital, Athens, Greece

**Keywords:** Pregnancy, Thyroid, Selenium

## Abstract

The management of pregnant women is a major concern of health care around the world. There is growing evidence regarding the influence of selenium (Se) on pregnancy and fetus outcomes. However, due to as yet insufficient evidence, lack of measurable markers to assess the effect of Se supplementation on the human metabolism, and Se’s narrow therapeutic index, the majority of experts and the current guidelines published by several scientific societies do not recommend the use of Se in pregnancy and in women of childbearing age. Further research based on well-designed studies, including assessment of the complex interactions between different micronutrients and individual response to different doses of Se, is needed.

## Introduction

The management of pregnant women to ensure an uneventful course of pregnancy and optimal fetal development is a major concern in health care practice.

Over the past few years, numerous clinical papers have been published which advocate pro or contra selenium (Se) supplementation during pregnancy [1]. However, the existing guidelines provided by several scientific societies and the majority of experts do not recommend the use of Se in pregnancy and in women of childbearing age due to as yet insufficient evidence [2].

In 2017, Ventura et al. presented in a review the current status of research in the field of Se and thyroid disease, indicating that in pregnancy, supplementation of Se appears to influence thyroid function and may be beneficial [1]. However, it was also underlined that in most studies, Se concentration was not measured prior to, during, or after supplementation, the most frequent primary measurements being of thyroid antibody levels. A very important issue is also Se’s narrow therapeutic index.

Nevertheless, there appear to be areas in which intervention with Se could potentially help to prevent such pregnancy complications as intrauterine growth restriction, miscarriage, preterm labor, preeclampsia, gestational diabetes, postpartum thyroiditis, and exacerbation of autoimmune thyroid diseases (AITDs) during the first postpartum year, and in the fetus, inter alia, decreased psychomotor development, neural tube defect, and deficits in the child’s cognitive development, intelligence, and behavior. The question remains, however, whether the abovementioned pregnancy complications are related to Se deficiency, and if so, how.

Se’s role in the physiology and pathophysiology of thyroid function is well known. Most of the enzymes involved in thyroid hormone synthesis contain Se, which, in the form of selenocysteine, is incorporated in a number of selenoproteins [3]. Thus, Se as a component of deiodinases is responsible for thyroid hormone activation, including conversion of prohormone T4 to its active form T3. In the brain, it takes part in antioxidant processes as an essential component of a number of enzymes, including glutathione peroxidases and thioredoxin reductases [4]. Se also plays a crucial role in the regulation of the immune response. The aim of this review was to present the current status of knowledge on the potential use of Se supplementation in pregnancy.

## Thyroid diseases in pregnancy

Hypothyroidism, thyroid autoimmunity, and hyperthyroidism are common in pregnancy. AITD carries a risk for impaired fertility and is the main cause of hypothyroidism in pregnant women, with the prevalence of its overt form being 0.50% and of its subclinical form (SCH) 3.47% [5], the estimates depending on the definition of the upper TSH cutoff value. Thyroid antibody positivity in childbearing women may be as much as 15–20%. Maternal hypothyroidism is related to such maternal complications as miscarriage and preeclampsia, and can lead to childhood cognitive impairment characterized by speech disorders, impaired motor development, and autism [2].

## Se and thyroid autoimmunity and fertility during pregnancy

The question of whether Se supplementation is justified in clinical practice in pregnant and childbearing women with AITD is an emerging issue. It has been hypothesized that since Se may act as an anti-inflammatory agent in autoimmune thyroiditis, women with this condition could potentially benefit from Se supplementation [1, 6]. In a randomized, placebo-controlled trial performed by Negro et al. in 2007, 77 and 74 TPOAb-positive pregnant women received 200 μg/day of selenomethionine and a placebo, respectively [6]. The authors found a reduction of TPOAb titres, an improvement of thyroid echostructure in ultrasound, and significantly less frequent postpartum thyroiditis, while there was reduced incidence of hypothyroidism in the subgroup receiving Se (28.6 vs 48.6% *p* < 0.01; 11.7 vs 20.3%, *p* < 0.01) [3]. It is worth mentioning that in most studies, neither Se concentration nor iodine status, which latter may also affect the effect of Se on thyroid function, were measured [1].

Residing in an area with relatively low Se intake increases the risk for a number of thyroid diseases, including subclinical hypothyroidism and AITD [1].

Recently, Ambroziak et al. published their results based on assessment of Se concentrations in a Polish population of pregnant women. Se status was assessed by serum Se and selenoprotein P (SELENOP) concentrations [7]. Low Se status and decreased Se and thyroid antibody concentrations throughout pregnancy were found. Se deficit (< 45 μg/l) was observed in 0%, 3.4%, 28.6% and 4.5%, 18.2% and 35.5% of women in the AITD and control group during the 1st, 2nd, and 3rd trimester, respectively [7]. The decline in TPOAb and TG-Ab was unrelated to Se status. In this study, TSH concentrations in newborns were also measured. No difference in average TSH concentration was found in children born to AITD mothers in comparison with healthy mothers (1.4 ± 1.4 mIU/l vs 1.8 ± 1.4 mIU/l). Additionally, there was no correlation between the TSH values in the newborns and serum Se or SELENOP concentrations of their mothers [7].

In 2017, the latest guidelines for diagnosis and management of thyroid diseases in pregnancy and postpartum were released under the auspices of the American Thyroid Association (ATA) [2]. The ATA guidelines do not recommend Se supplementation in pregnancy due to the conflicting literature data, which render any generalized recommendation unreliable. Among other data coming mainly from nonpregnant women, reduction of thyroid antibodies due to Se supplementation was not observed [8–11]. Moreover, some authors found a correlation between Se supplementation and a higher risk of developing other diseases, such as type 2 diabetes mellitus [12]. The ATA underlined the need to take into account different intakes of iodine, Se, or both in different regions before any action is undertaken regarding Se supplementation.

Antioxidant supplementation, including Se in pregnancy, may have a significant impact on pregnancy outcome and child neurodevelopment [1, 2, 13–15]. In the two-armed randomized trial performed in Iran with 100-μg Se vs placebo applied in the first trimester, Tara et al. reported lower incidence of preeclampsia and premature rupture of membranes in the group receiving Se [13, 14]. It was also found that a low blood serum concentration of Se in the early stage of pregnancy could be a predictor of low birth weight in newborns [15].

In another prospective cohort study performed in Bangladesh, the impact of Se status in pregnancy on child development at 1.5 years of age was evaluated [16]. In 750 mothers, Se status was assessed at 30 gestational weeks and erythrocyte Se (Ery-Se) was measured. Ery-Se is considered the most suitable biomarker of long-term Se status assessment (expressed as μg/g Hb). Children of the mothers recruited into the study underwent psychological testing. A revised version of the Bayley Scales of Infant Development and a Bangladeshi version of MacArthur’s Communicative Development Inventory were used to assess children’s mental and psychomotor development and their language comprehension and expression [16]. Various micronutrient supplements were administered at gestational week 14 (65 μg of Se as sodium selenite). Maternal Ery-Se varied from 0.19 to 0.87 μg/g Hb and correlated with childhood developmental measures. The authors noted that an increase of Ery-Se by 0.50 μg/g Hb was associated with an increase in children’s language comprehension by 3.7 points as well as corresponding to an increase in girls’ psychomotor development by 12 points (by much fewer points in boys). They thus concluded that a low prenatal Se status seems to be disadvantageous for children’s psychomotor and language development.

In a study carried out in Poland, Polanska et al. also demonstrated a significant relationship between motor development at 1 year of age and language development at 2 years and the mothers’ Se status, while noting borderline significance as concerns cognitive score assessment [17].

Several interventional studies have been performed, mainly with low doses of Se. In the double-blind, randomized, placebo-controlled longitudinal SPRINT (Selenium in PRegnancy INTervention) study, 60 μg/day of Se was given from pregnancy week 12 until delivery. The different possible associations between Se intake and potential effects were analyzed. No worsening of insulin resistance and development of type 2 diabetes mellitus was found [18]. Further analysis showed that Se potentially reduces the frequency of pregnancy-induced hypertension [19] and decreases serum soluble vascular endothelial growth factor receptor-1 related to the risk of preeclampsia [20], but no effect on TPOAb was observed [21].

While the effect of Se administration should be considered in relation to its potential benefits in individuals with Se deficiency, it may pose a risk in people with excess Se intake. Se supplementation added to L-thyroxin can be beneficial in patients with low intake of this micronutrient and with a mild form and/or early stage of AITD. Serum Se concentration decreases in inflammation and may vary depending on the severity and duration of the inflammatory process

AITD is linked to abnormal folliculogenesis and embryogenesis [22–25]. Alterations of the local activity of thyroid hormones and their impact on the regulation of the abovementioned processes may play an essential role in early pregnancy and pregnancy loss [23]. Se was also found to be an important microelement involved in migration and proliferation processes in trophoblasts where it acts by reducing mitochondrial oxidative stress. According to a recently performed study on cell lines, there are indications that Se may be used in reproductive medicine for such purposes as infertility treatment [26]. However, further data are necessary to confirm these results.

## Se sources and daily requirement in pregnancy

In humans, diet is the main source of Se, with intake depending mainly on soil content. In 2019, the European Food Safety Authority published data regarding diet Se requirements. Recommended Se intake is 60–70 μg/day for adults, with increased requirements for pregnant and lactating women, i.e., 70 and 80 μg/day, respectively [27]. Supplementation with the organic form is likely to be more effective.

Intake of Se varies widely globally, with, for example, a high intake in Venezuela, Canada, the USA, and Japan, and a much lower intake in Europe [28]. Meanwhile, the fact that selenium-enriched dietary supplements are nowadays on the increase worldwide, particularly among pregnant women, makes it more difficult to assess Se intake in many populations. The total content of Se may vary significantly in the different series of a given supplement, as well as in the various supplements. Almeida et al. compared the amount of Se in eight commercially available food supplements and found that the minimum was 15.4 ± 0.9 μg/unit, whereas in several others it was as high as 205.3 ± 9.9 μg/unit [29]. Generally speaking, the public needs to be aware of the potential health risk of taking Se supplements, but this is especially true for future mothers, since these supplements can have adverse effects on both the mother and the developing fetus. Determination of Se in food supplements is of major importance due to the low range between the beneficial and toxic effects of this microelement, thus, a policy to control Se supplements in all countries is urgently needed.

Because the current knowledge on microelements and their very diverse interactions in the body is limited, there has been to date no analysis of the role of a single microelement such as Se in pregnancy. An additional complication is the fact that there are multiple environmental factors that can trigger an autoimmune response in genetically susceptible individuals [1, 2, 11].

Although the exact mechanisms linking environmental factors to thyroid autoimmunity are not as yet fully understood, there is increasing evidence that the following environmental factors are involved in development of autoimmune diseases: environmental pollution (e.g., organochlorines and pesticides), nutrients with iodine, Se, iron, zinc, vitamin B12, and D, irradiation, drugs (interferon α and γ, TNF-α), smoking, obesity, socioeconomic status, infections, stress, lifestyle, and pregnancy [30]. Recently published data from Australia showed that lower plasma copper measured at pregnancy week 15, even though adjusted to Se and zinc status, had decreased the risk of any pregnancy complications [31]. The authors concluded that further research to determine the role of trace elements in early pregnancy should specifically focus on their interactions in supporting fetal development and pregnancy outcome.

Increased Se requirements during pregnancy may deteriorate Se status. Recently, the important role of genetic predisposition with the genotype of the Se transport protein is underlined [17, 32]. It has been observed among European pregnant women that Se deficiency can exacerbate mild iodine deficiency, which is a common disorder in Europe [32].

Se intake in the lactating mother and its content in breast milk are closely intertwined. The amount of Se in maternal milk and its bioavailability is higher compared with that in cows’ milk formula. The suboptimal level of Se in human milk was observed in different European geographical areas [33–35]. In Poland, for example, the calculated daily Se intake by breast-fed infants was below the recommended amount, ranging from 6.46 to 8.50 μg/day, depending on the mothers’ place of residence [35].

## The potential role of selenium supplementation in pregnancy

Se, as mentioned above, is a component of selenoproteins, which are crucial for thyroid hormone synthesis and immune response regulation. Based on the latter, before the drawing up of Se supplementation recommendations, conclusive results are required from recently published well-designed research and/or ongoing studies. Nevertheless, in a study performed in the UK on pregnant women residing in an area with mild to moderate iodine deficiency, Mao et al. explored the effect of low-dose Se supplementation on thyroid function and peroxidase antibody concentration. Se intervention was at 12–14 weeks of pregnancy. Two hundred and thirty women were randomized into two arms, 60 μg/day of Se and placebo. No effect of Se supplementation on TPOAb was observed, but a tendency for thyroid function changes was noted [20].

An analysis of the association between maternal Se concentrations and child neuropsychological development was performed in the first phase (2003-2005) of the Spanish Childhood and Environment Project (INMA) [36]. Six hundred and fifty mother-child pairs were enrolled in the study. Se concentration measured at 3 months of pregnancy was 79.7 ± 7.9 μg/L. The authors observed an inverted U-shaped (non-linear) relationship between maternal Se concentrations and child neuropsychological development assessed with the use of the Bayley Scales of Infant Development (BSID) performed at 12 months of age. Additionally, if Se concentration was higher than 86 μg/L, a decreasing association between BSID scores and maternal Se status was found. The authors suggested that the fetus could be vulnerable to Se neurotoxicity at these levels. Additionally, the results were related to the Se metabolic patterns based on child indolethylamine N-methyltransferase (INMT), AG+AA genotype for rs6970396. A descending curve was proposed for the GG genotype, confirming the important role of individual susceptibility [36]. It is worth mentioning that there is a large variability in the metabolism of Se and in the urinary excretion of its metabolite trimethylselenonium ion (TMSe), and this variability has been strongly linked to a genetic variation of the INMT gene [37]. However, it is unclear whether the INMT polymorphism may also modify the effect of Se in children.

A recent study also conducted by Amoros and colleagues based on the same patient cohort, with 490 mother-child pairs taking part, showed consistency in the results. The neuropsychological development of the children was assessed at 5 years of age (INMA, 2003–2012) using the McCarthy Scales of Children’s Abilities (MSCA) [38]. Four hundred and ninety mother-child pairs took part in the study. Medium maternal Se concentration was similar to the previous levels, namely, 79.9 ± .8.1 μg/L. Similarly, generalized additive models indicated inverted U-shaped relationships between Se concentrations and the children’s verbal and global memory scales, while also similar was the finding that their results worsened with Se concentrations higher than 85 μg/L. All models were adjusted for sex, maternal age, maternal country of birth, maternal educational level, parity, type of zone, maternal working status during pregnancy, and BMI before pregnancy. Thus, the results derived from the INMA study suggested a nonlinear relationship between Se concentrations and children’s neurodevelopment. Interestingly, the protective effect of breastfeeding was most clearly observed with low values of maternal Se levels. Ultimately, the main conclusion from both phases of the study is the same: low and high maternal Se concentrations seem to be harmful for a child’s neuropsychological development. Se concentrations in this population were within the range considered to be safe for human health, which implies that prenatal and early life periods could be especially vulnerable to Se neurotoxicity.

A similar observation came some years ago from a study conducted in China in 2013, specifically, that both low and high levels of cord serum Se were inversely associated with neurobehavioral development among newborns [39].

Se concentrations varied markedly in different studies, a possible reason for the conflicting results obtained, particularly those of earlier published papers. Future studies should be designed in such a way as to closely investigate how micronutrients act in combination with other factors and the intricate interactions between them.

## Are we ready to implement Se supplementation in pregnancy?

The question whether, on the basis of the current evidence, Se supplementation should be implemented in pregnancy can at present only be replied to in the negative. Arguments for and against Se supplementation in pregnant women are summarized in Table 1.

**Table 1:** Arguments for and against Se supplementation in pregnant women

Pro	Contra
• Maternal nutrient status can influence development of fetal brain • Common TPOAb positivity • Diminishment of risk of known AITD pregnancy complications, such as miscarriage and preterm delivery • Possible prevention of PPTD and permanent hypothyroidism by the administration of Se during pregnancy: only 1 study	• The rather narrow Se therapeutic range and the risk associated with overdosage • Se has no proven benefit among patients planning pregnancy in terms of thyroid function and TPOAb titers • The lack of clear-cut benefits in cases of thyroid autoimmunity and pregnancy and the potential side effects of Se supplementation

*TPOAb* thyroid peroxidase antibodies, *AITD* autoimmune thyroid diseases, *PPTD* postpartum thyroid diseases

Se serum concentration and its content in Se-containing proteins are not directly linked to thyroid Se content and activity of selenoproteins in the thyroid gland [22]. Moreover, since, according to the literature, no measurable markers exist to assess unequivocally and reliably the effect of Se supplementation on the human metabolism [40, 41], more data are required with regard to Se supplements, particularly when this concerns the wellbeing of the mother and of the developing fetus.

In 2016, Negro et al. published the results of a survey performed among Italian doctors regarding the use of Se in pregnant women [42]. Despite the fact that such a recommendation does not at present exist, 40% of respondents (778 completed questionnaires) supported Se use mainly in order to prevent development of hypothyroidism and postpartum thyroiditis, as well as to reduce TPOAb titers. Thirty percent of doctors suggested less than 100 μg of Se daily and 60% 100–200 μg/day, while 10% recommended more than 200 μg/day. The authors concluded that physicians should be more prudent in advocating Se supplementation among women of childbearing age or pregnant women with AITD and need to be aware of the possible toxic effects of Se [42].

Recently, Morrill, summarizing the data derived from all the trials conducted to date on Se, indicated that Se supplementation during pregnancy is often associated with several health benefits, such as reduced incidence of pregnancy-induced hypertension, preeclampsia, premature rupture of membranes, postpartum depression, and decreased biomarkers of oxidative stress while having no adverse effect on glucose metabolism [43]. He reports that 100–200 μg/day of Se appears to be safe and could be considered, provided that regional differences in diet are taken into account. Daily Se intake should not exceed 400 μg in total (in conformity with the Institute of Medicine, Food and Nutrition Board statement).

It is universally known that healthy eating during pregnancy and lactation is essential to ensure a “healthy” pregnancy and the wellbeing of offspring. Though some authors have recently proposed the translation of nutrient recommendation into the personal diet of pregnant and lactating women [44], currently, in all medical procedures, tailored therapy and personalized diagnostics is recommended as being the optimal approach.

## Summary

There are some literature data demonstrating potential benefits of Se supplementation in pregnancy. However, due to a lack of sufficiently sensitive markers assessing Se homeostasis in the human organism, as well as the scant knowledge about the complex interactions between different micronutrients and the as yet unknown individual response to different doses of Se, recommendation for Se supplementation is not currently possible. There is thus a pressing need for further research along with well-designed and adequately powered trials before any steps toward Se supplementation in pregnancy may be taken.
